# Infection-specific PET imaging with ^18^F-fluorodeoxysorbitol and 2-[^18^F]F-ρ-aminobenzoic acid: An extended diagnostic tool for bacterial and fungal diseases

**DOI:** 10.3389/fmicb.2023.1094929

**Published:** 2023-01-25

**Authors:** Marta Rua, Jon Ander Simón, María Collantes, Margarita Ecay, José Leiva, Francisco Carmona-Torre, Rocío Ramos, Félix Pareja, Krishna R. Pulagam, Jordi Llop, José Luis Del Pozo, Iván Peñuelas

**Affiliations:** ^1^Clinical Microbiology Laboratory, Clínica Universidad de Navarra, Pamplona, Spain; ^2^Instituto de Investigación Sanitaria de Navarra (IdiSNA), Pamplona, Spain; ^3^Radiopharmacy Unit, Department of Nuclear Medicine, Clinica Universidad de Navarra, Pamplona, Spain; ^4^Translational Molecular Imaging Unit, Department of Nuclear Medicine, Clinica Universidad de Navarra, Pamplona, Spain; ^5^Infectious Diseases Division, Clínica Universidad de Navarra, Pamplona, Spain; ^6^Basque Research and Technology Alliance (BRTA), CIC BiomaGUNE, San Sebastián, Spain

**Keywords:** FDG (18F-fluorodeoxyglucose)-PET/CT, PABA para-aminobenzoic acid, 18[F]FDS, 18[F]FPABA, mouse model, PET imaging, sorbitol, folate

## Abstract

**Introduction:**

Suspected infectious diseases located in difficult-to-access sites can be challenging due to the need for invasive procedures to isolate the etiological agent. Positron emission tomography (PET) is a non-invasive imaging technology that can help locate the infection site. The most widely used radiotracer for PET imaging (2-deoxy-2[^18^F] fluoro-D-glucose: [^18^F]FDG) shows uptake in both infected and sterile inflammation. Therefore, there is a need to develop new radiotracers able to specifically detect microorganisms.

**Methods:**

We tested two specific radiotracers: 2-deoxy-2-[^18^F]-fluoro-D-sorbitol ([^18^F]FDS) and 2-[^18^F]F-ρ-aminobenzoic acid ([^18^F]FPABA), and also developed a simplified alternative of the latter for automated synthesis. Clinical and reference isolates of bacterial and yeast species (19 different strains in all) were tested *in vitro* and in an experimental mouse model of myositis infection.

**Results and discussion:**

Non-lactose fermenters (*Pseudomonas aeruginosa* and *Stenotrophomonas maltophilia*) were unable to take up [^18^F]FDG *in vitro*. [^18^F]FDS PET was able to visualize Enterobacterales myositis infection (i.e., Escherichia coli) and to differentiate between yeasts with differential assimilation of sorbitol (i.e., *Candida albicans* vs. *Candida glabrata*). All bacteria and yeasts tested were detected *in vitro* by [^18^F]FPABA. Furthermore, [^18^F]FPABA was able to distinguish between inflammation and infection in the myositis mouse model (*E. coli* and *Staphylococcus aureus*) and could be used as a probe for a wide variety of bacterial and fungal species.

## 1. Introduction

Infectious diseases have become a major problem, with an increasing incidence, pandemic scenarios, and the spread of antibiotic resistance (Morens and Fauci, 2020). Diagnosis is usually based on clinical signs and symptoms, laboratory analyses, microbiological tests, and imaging tools. Conventionally, the main laboratory techniques used to identify bacteria and fungi are culture methods, which are accurate but very time consuming and can take several days or even weeks. Furthermore, culture methods sometimes require invasive sampling procedures that can involve risks at the time of collection.

[^18^F]FDG is a PET radiotracer that is widely used in the diagnosis and follow-up of tumors and degenerative diseases of the central nervous system (Dubois et al., 2014; Rowe and Pomper, 2021; Guedj et al., 2022). Conventional *in vivo* nuclear imaging using radiolabeled monocytes (Chakfé et al., 2020; Erba and Slart, 2020) or positron emission tomography (PET) with 2-deoxy-2[^18^F]fluoro-ᴅ-glucose ([^18^F]FDG) can provide a rapid diagnosis of difficult-to-diagnose infections (endocarditis, vascular graft infection, and prosthetic joint infection) and at the same time avoid invasive sampling procedures (Kwee et al., 2008; Habib et al., 2015; Mahmood et al., 2019; Haidar and Singh, 2022; Lauri et al., 2022). [^18^F]FDG is an extensively available radiotracer that can be obtained from a cyclotron center and shipped to the hospital because of the long half-life of ^18^F (110 min). However, nonspecific uptake at sterile inflammation sites and the inability to differentiate between different microorganisms can however yield ambiguous results (Auletta et al., 2019).

The narrative evidence has expressed the need for PET tracers to be able to selectively detect microorganisms. 2-deoxy-2-[^18^F]-fluoro-ᴅ-sorbitol ([^18^F]FDS) is a fluorinated probe that specifically detects Enterobacterales (Weinstein et al., 2014; Yao et al., 2016; Ordonez et al., 2021), although infectious diseases caused by Gram-positive bacteria and fungi may be underdiagnosed. [^18^F]FDS is a fluorine-18 analog of sorbitol that is easy to synthesize from [^18^F]FDG. The first study conducted with [^18^F]FDS focused on molecular imaging of brain tumors (Li et al., 2008). However, the active sorbitol transporter (the PTS = phosphoenolpyruvate:glycose phosphotransferase system) and metabolism are specific to certain GN bacilli (i.e., Enterobacterales). Sorbitol is a sugar that requires PTS-mediated uptake into the bacterial cell (Cordaro, 1976). Phosphorylation prevents the [^18^F]FDS from escaping, and so the probe accumulates within the cell. The PTS is essential for bacterial uptake of [^18^F]FDS, but not for [^18^F]FDG (Ordonez et al., 2021). This transporter specificity defines the sorbitol-analog as a class-specific bacterial probe.

In an analysis to find pathogen-specific imaging tracers, para-aminobenzoic acid (PABA) derivatives were identified as potentially suitable tracers for all bacteria (Ordonez et al., 2017). The folate pathway is a key component in DNA and amino acid biosynthesis. Folate biosynthesis is essential in bacteria, yeasts, protozoa, and plants. By contrast, mammalian cells are unable to carry out *de novo* synthesis using this pathway and so need to acquire folate in the diet. PABA is the substrate for an essential enzyme [dihydropteroate synthase (DHPS)] in this process. DHPS catalyzes PABA and a pterin pyrophosphate to produce dihydropteroate (Bermingham and Derrick, 2002). Previous studies have concluded that the fluorinated probe of PABA is an alternative substrate for *Staphylococcus aureus* DHPS that rapidly accumulates in the bacteria (Zhang et al., 2018). Fluorine-18 labeled tracer, 2-[^18^F]F-ρ-aminobenzoic acid ([^18^F]FPABA), was used to detect *S. aureus* myositis infection and monitor the efficacy of antibiotic treatment in a rat model of myositis (Zhang et al., 2018). [^11^C]PABA was tested in human volunteers with no adverse effects (Ordonez et al., 2022), although one of the drawbacks of carbon-11 is the extremely short half-life for radionuclide decay (20.4 min) which would rule out the distribution of [^11^C]PABA over long periods of time. In addition, metabolism of the carbon-11 moiety could also lead to image distortions, or even require plasma metabolism analysis, an additional challenge for carbon-11-labeled tracers. For these reasons, [^18^F]FPABA could be a better choice for the development of PET infection radiotracers.

Molecular imaging tracers are mainly developed and tested only for bacterial infections. Fungal infections are rarely considered, although they represent potentially life-threatening opportunistic diseases in certain patients (such as the critically ill and/or immunosuppressed). Invasive fungal infections (IFIs) caused by *Candida* spp. are a leading cause of infection in patients with underlying hematopoietic stem cell transplantation indications, solid organ transplant recipients, and the critically ill (Jenks et al., 2020). Disseminated candidiasis may involve the central nervous system (CNS; Góralska et al., 2018), and [^18^F]FDG PET imaging reveals high glucose metabolism in the CNS, making diagnosis challenging. In another study, [^18^F]FDG PET/CT was of added value as a diagnostic tool for IFIs in the management of 74% of patients (Ankrah et al., 2021). Another clinically relevant fungus (*Aspergillus fumigatus*) was evaluated with [^18^F]FDS without investigators supporting its use (Lai et al., 2022). However, the most recent study on [^18^F]FDS uptake in *Aspergillus fumigatus in vivo*, in a mouse model, indicated that visualization of infection in the lungs, brain and muscles was possible (Kim et al., 2022). No preclinical studies or experimental animal models have evaluated PABA-labeled radiotracers in yeast or filamentous fungi.

In this study, we tested the *in vitro* uptake of [^18^F]FPABA, [^18^F]FDS, and [^18^F]FDG in multiple species of bacteria and yeast obtained from reference (American Type Culture Collection: ATCC) and clinical isolates (prosthetic infections). We also compared the *in vivo* uptake of these radiotracers in an acute myositis model with representative Gram-positive (GP) and Gram-negative (GN) bacteria. Given that the currently available [^18^F]FPABA radiotracers are complex and cumbersome, we also aimed to develop a simplified alternative for automated synthesis of this radiotracer that would make it more widely available, using standardized procedures amenable to Good Manufacturing Practices (GMP) compliance. This approach brings innovative, pathogen-specific imaging tracers closer to clinical settings and tests the actual diagnostic use of [^18^F]FDG by analyzing multiple bacterial species.

## 2. Materials and methods

### 2.1. Synthesis of [^18^F]FDG, [^18^F]FDS, and [^18^F]FPABA

Synthesis of [^18^F]FDG was by standard nucleophilic substitution using an IBA Synthera module (IBA, Louvaine-la-Neuve, Belgium). [^18^F]FDS was synthesized following previously described methods, with some modifications (Weinstein et al., 2014; Li et al., 2017). Briefly, [^18^F]FDG was reduced with 2 mg sodium borohydride at 35°C for 30 min with periodic shaking, the reaction was stopped by the addition of 1.4 ml of a sodium acetate/HCl solution (0.9/0.4 M), and the final pH was adjusted to 7 with NaOH. The final product was purified using an Alumina N Light Sep-Pack cartridge and sterile filtered (0.22 μm). Radiochemical purity was >97%, as determined by radio-thin layer chromatography (radio-TLC).

**Figure 1:**
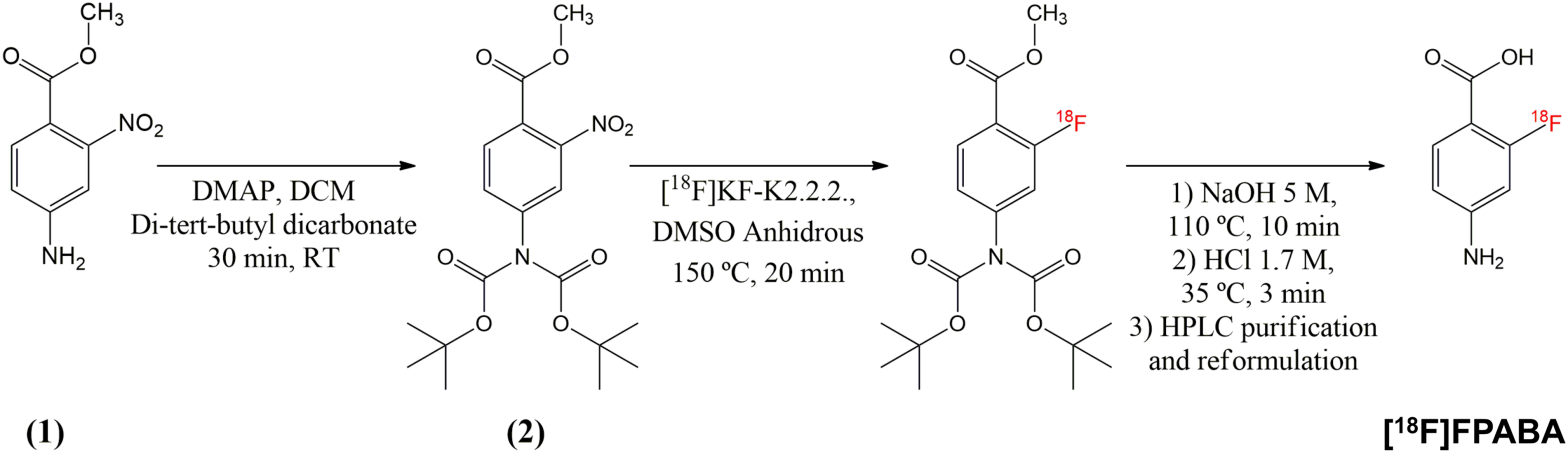
Synthesis pathway of [^18^F]FPABA from commercially available methyl 2-nitro-4-aminobenzoate. Compound **(2)** was used as the precursor for the automated radioactive synthesis.

**Figure 2:**
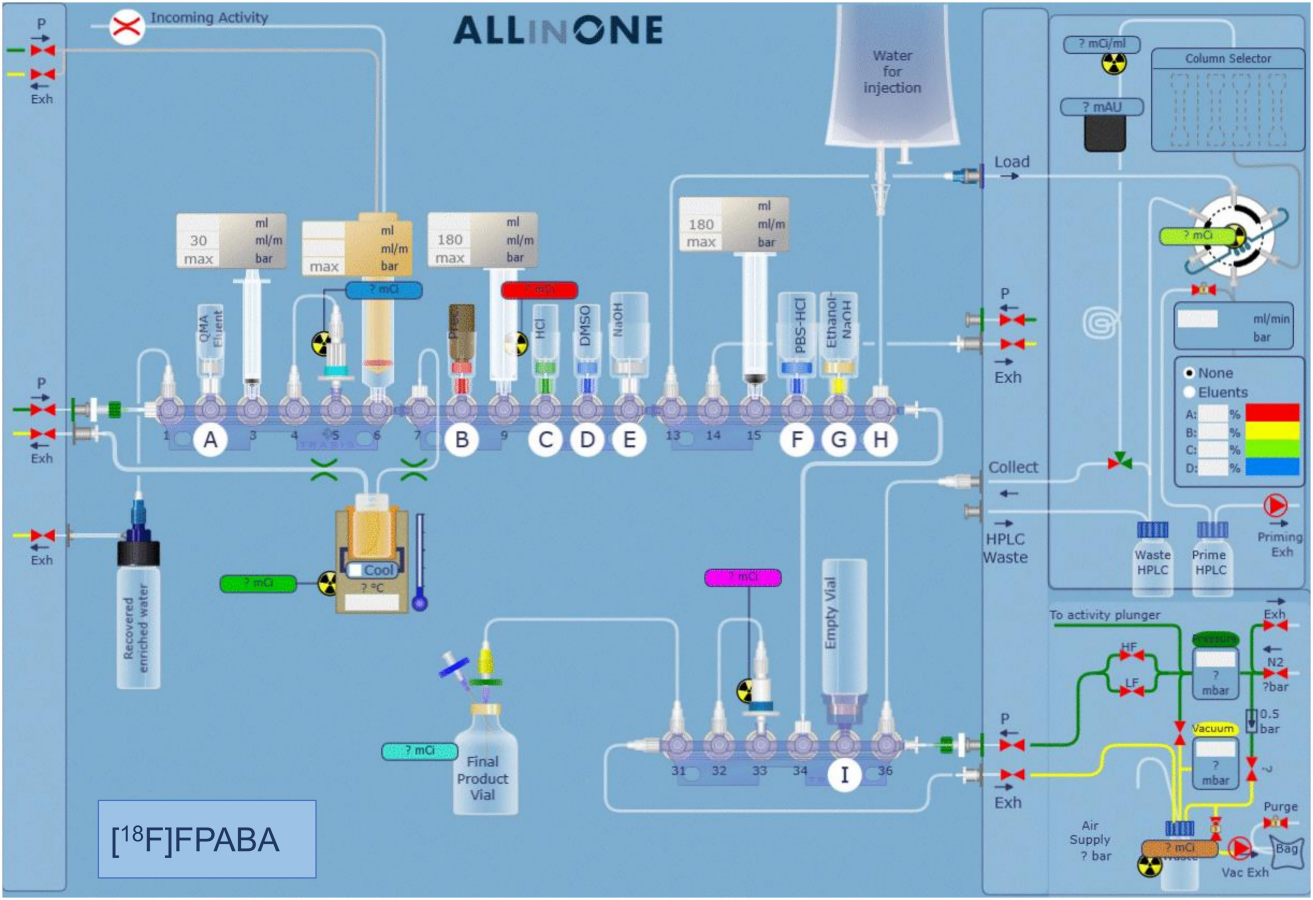
Automated synthesis software interface showing the position of the different reagents and systems used for the synthesis. The various steps of the synthesis are described in detail in the methodology section.

The available synthesis methods of [^18^F]FPABA (Zhang et al., 2018; Li et al., 2020) are complex and require a precursor that is difficult to prepare. Consequently, we prepared a new precursor [methyl 4-*N*,*N*-di(Boc)-2-nitro-4-aminobenzoate **(2)**] from a commercially available reagent to enable a one-step nucleophilic substitution synthesis in an automated synthesis module (Figure 1). To a solution of methyl 2-nitro-4-aminobenzoate **(1)** (0.25 g, 34 mmol) and di-tert-butyl dicarbonate (7.4 g, 68 mmol) in methylene chloride (10 ml), cooled with ice water, was added one equivalent of 4-(dimethylamino)pyridine. After stirring for 30 min at room temperature (RT), the mixture was diluted with an additional 50 ml of methylene chloride, washed with brine, and dried over MgSO_4_. Removal of the solvent gave 370 mg of the crude product, which was purified by silica gel column chromatography using CH_2_Cl_2_/methanol (95/5) as eluent to yield 290 mg (58%) of **2** as a white solid. For the radiosynthesis, [^18^F]F^−^ produced by irradiation of H_2_^18^O in a 18/9 cyclotron (IBA, Belgium) was trapped on a QMA cartridge and eluted with 0.5 ml of K_2_CO_3_/Kryptofix 2.2.2. After azeotropic drying, 2 mg of precursor **2** was added to the reactor in 1 ml *N*, *N*-Dimethylformamide, heated to 150°C for 20 min, hydrolyzed at 110°C for 10 min with 0.5 ml of 5 M NaOH, and then cooled to 40°C and neutralized with 1.5 ml of 1.7 M HCl. The crude product was purified by radio-HPLC using a VP 250/16 Nucleosil 100–7 C18 column, and 90/10 trifluoroacetic acid (TFA) 0.1% in H_2_O/MeCN as the mobile phase. [^18^F]FPABA (eluted at 9–10 min) was collected in 40 ml of water, reformulated by trapping on a MCXplus cartridge, and eluted with 2 ml of ethanol/NaOH 1 M (50/50) into a citrate buffer solution to a final volume of 5 ml. All radiosynthesis steps took place in an automated synthesis module (AiO36, Trasis, Belgium), for which a specific program and user interface (Figure 2) were developed.

Radiochemical purity was determined by HPLC on a Mediterranea Sea C18 (150 × 4.6) column with a 0.1% TFA/MeCN gradient at 1 ml/min. [^18^F]FPABA was eluted at 5.7 min, whereas the free [^18^F]F^−^ was eluted at 2 min.

### 2.2. *In vitro* uptake assays

The following bacterial and fungal reference strains (ATCC) were used: *Staphylococcus epidermidis* ATCC 12228 and ATCC 35984, *S. aureus* ATCC 29213 and 25923, *Cutibacterium acnes* ATCC 11827, *Escherichia coli* ATCC 25922, and *Pseudomonas aeruginosa* ATCC 27853 and *Candida albicans* ATCC 10231. Selected clinical isolates from patients with prosthetic material were also included (Supplementary Table S1).

All microorganisms, except *C. acnes,* were cultured the night before on solid agar, followed by 20–24 h of culture in tryptic soy broth (TSB, bioMérieux) at 37°C under constant agitation. *Cutibacterium acnes* was cultured for 48 h on solid agar, then for another 48 h in thioglycolate broth (TG, bioMérieux) under anaerobic conditions. Broth medium was selected to achieve better tracer uptake compared to a solid media. In addition, a prolonged incubation time was set to allow the microorganisms to consume most of the D-glucose.

The next day, the incubation broth was diluted in 4 ml 0.9% NaCl and adjusted with more incubation broth or saline until an optical density of 0.75 McFarland units was reached. Dilution in saline was chosen to reduce the amount of D-glucose in case any traces remained that might be available and compete with the carbohydrate tracers ([^18^F]FDG and [^18^F]FDS). Three replicates (500 μl) of each microorganism were incubated with radiotracers (100 μl, 37 ± 11 MBq/ml) for 2 h under agitation, pelleted by centrifugation, and washed with PBS. Final radioactivity in all pellets was measured using a gamma counter (Hidex Automatic Gamma Counter) calibrated for fluorine-18 and normalized to the initial number of colonies (Bq/10^6^ CFU: colony-forming units). Aliquots of *E. coli* ATCC 25922 with the same optical density were used as negative control after inactivation at 90°C for 30 min. Counts per minute were corrected for background and decay. The experiment was repeated three times on each strain in three independent experiments, using triplicate of PBS as background.

The number of viable microorganisms was quantified at the beginning and end of each experiment. CFU per ml (CFU/ml) values were determined by dilutions and plating on trypticase soy agar (TSA) under optimal growth conditions depending on the microorganism. Initial CFUs were measured to normalize to activity (Bq/10^6^ CFU) and as a control. The number of colonies in the final pellet was measured to monitor the growth of the microorganisms.

### 2.3. Mouse myositis infection model

All procedures involving animals were carried out in accordance with the guidelines of the European Communities Council Directive (2010/63/EU) and the Spanish Government (RD 53/2013) and were approved by the Animal Experimentation Ethics Committee of the University of Navarra (Protocol no. 103-17). Four-week-old male and female ICR mice (*n* = 42; males = 16 and females = 26) were purchased from Envigo and socially housed in the animal facilities of the University of Navarra under controlled conditions (22 ± 2°C, 12-h light/12-h dark cycle; relative humidity 55 ± 10%).

*Staphylococcus aureus* ATCC 29213 and *E. coli* ATCC 25922 were selected as representative GP and GN bacteria to develop the mouse model of acute myositis.

Fresh bacterial strains were incubated in TSB broth overnight. Inoculated bacteria were diluted and incubated for an extra hour to obtain exponential growth. Bacterial load was adjusted to 7–8 log_10_ CFU. 50 μl of live bacteria were injected into the right hindlimb of the animals; the same volume of heat-inactivated inoculum (90°C, 30 min) was injected into the left hindlimb to simulate sterile inflammation. The mice were imaged with [^18^F]FDG, [^18^F]FDS, or [^18^F]FPABA 16 to 18 h after the initial infection for comparison.

#### 2.3.1. PET/CT imaging

All PET images were acquired on a dedicated small animal Mosaic tomograph (Philips) and reconstructed, applying dead time, decay, random, and scatter corrections, into a 128 × 128 matrix with voxel size of 1 mm. Computed tomography (CT) scans were also performed in U-SPECT6/E-class (MILabs) imaging equipment to obtain the corresponding anatomical images, using a tube setting of 55 kV and 0.33 mA.

For PET imaging, the mice were fasted overnight with *ad libitum* access to drinking water. On the day of the study, radiotracer was injected intravenously *via* a tail vein ([^18^F]FDG: 9.2 ± 0.2 MBq; [^18^F]FDS: 9.3 ± 1.1 MBq; [^18^F]FPABA: 13.5 ± 5.2 MBq). The mice were kept anesthetized with 2% isoflurane in 100% O_2_ gas after administration of the radiotracer. One hour after injection of [^18^F]FDG or [^18^F]FPABA (2 h for [^18^F]FDS), the animals were placed prone on the scanner bed for a 15-min image acquisition. Twenty minutes beforehand, the animals received an intravenous injection of 100 μl furosemide (10 mg/ml) and 1.5 ml of subcutaneous saline solution (0.9%).

All studies were exported and analyzed using PMOD software v 4.105 (PMOD Technologies Ltd., Adliswil, Switzerland) and converted into standardized uptake value (SUV) units using the formula SUV = [tissue activity concentration (Bq/cm^3^)/injected dose (Bq)] × body weight (g). The PET images were registered with their corresponding CT scans to localize the uptake signal. A semi-quantitative analysis was performed by manually drawing volumes of interest (VOI) containing signal at the site of infection or inflammation. After obtaining the mean uptake value in each VOI, the ratio of values for infected or inflamed hindlimbs was calculated (SUVr).

#### 2.3.2. *Post-mortem* measurement of radioactivity uptake and microbiological analysis of hindlimbs

After euthanasia, the infected and inflamed limb muscles were harvested and weighed. The ^18^F radioactivity of each sample was measured with a gamma-counter. ^18^F uptake in infected and inflamed hindlimbs was presented as percentage of injected dose per gram of tissue (%ID/g).

Colony-forming units were quantified by homogenization in PBS and growth on TSA medium using dilutions, and in BHI broth (brain heart infusion) incubated at 37°C with 10% CO_2_ for at least 2 days. The concentration of bacteria was measured as CFU/g of muscle.

### 2.4. Statistical analysis

For data analysis, the Mann–Whitney U test was used to compare two groups, and the Kruskal Wallis test for multigroup comparison. Multigroup mean comparisons of *in vitro* growth at the beginning and the end of experiments (CFU/g) were analyzed with Sidak’s test. Data are shown as mean ± SD.

## 3. Results

### 3.1. [^18^F]FDS synthesis and automated [^18^F]FPABA synthesis

The [^18^F]FDS synthesis procedure yielded a high purity product (>97%) over 20 different syntheses. The most suitable TLC chromatography method for analysis was the one used for [^18^F]FDG following European Pharmacopeia instructions (01/2014:1325). The final product always appeared as a sharp peak at a retention factor (Rf) of 0.15–0.20, while the precursor appeared at over 0.5. We checked that the final pH of the formulation was 7.0 ± 0.5 and adjusted with the corresponding acid or base if necessary.

The precursor, methyl 4-*N*,N-di(Boc)-2-nitro-4-aminobenzoate (used for radiosynthesis of [^18^F]FPABA), was prepared in a one-step reaction with an average yield of 58%. The implementation of radiosynthesis in the automated system (TRASIS AllinOne 36 module) proved to be reliable, reproducible, and amenable to GMP implementation. Overall, radiosynthesis took less than 80 min (including radio-HPLC purification and reformulation) and [^18^F]FPABA was produced with a radiochemical yield of 6% (final activities were in the range of 1.3–1.8 GBq in 4 ml) and > 95% radiochemical purity, as assessed by radio-HPLC (Supplementary Figure S2). [^18^F]FPABA obtained a specific activity of 395.53 ± 189.07 GBq/μmol at the end of synthesis. The injectable solution of [^18^F]FPABA had a pH of 6, contained 10% ethanol as a stabilizing agent and was stable for at least 8 h as determined by radio-HPLC.

### 3.2. *In vitro* uptake by reference and clinical strains

**Figure 3:**
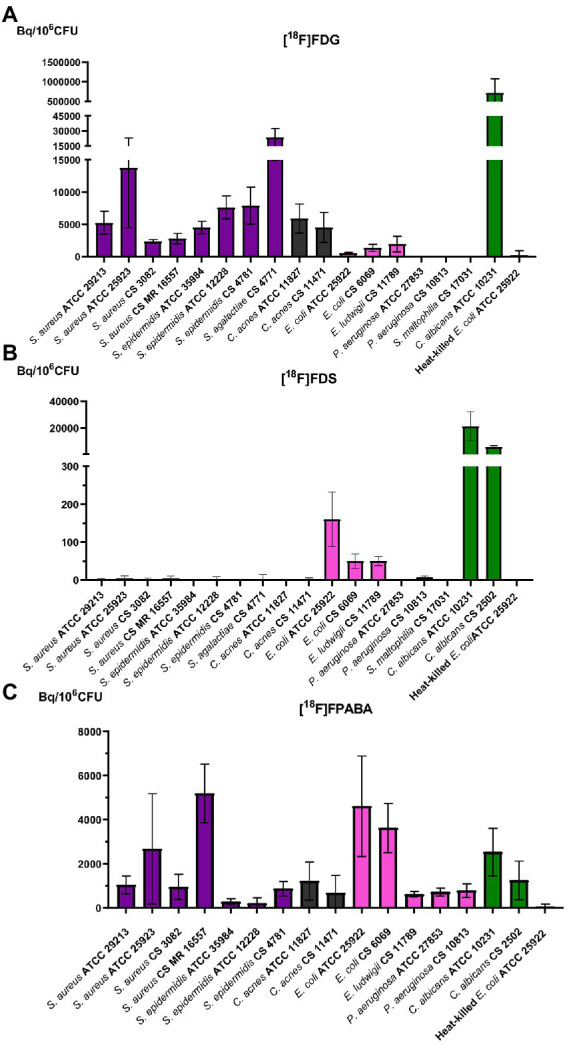
Cellular uptake of different probes tested after 2 h of accumulation. Purple: Gram-positive bacteria. Gray: *Cutibacterium acnes*. Pink: Gram-negative bacteria. Green: yeasts. CS: clinical strain. **(A)** [^18^F]FDG; **(B)** [^18^F]FDS; and **(C)** [^18^F]FPABA.

All bacteria except *Pseudomonas aeruginosa* and *Stenotrophomonas maltophilia* incorporated [^18^F]FDG (Figure 3A). [^18^F]FDG uptake in GP bacteria was higher than in GN bacteria. Among Gram-positives, *Streptococcus agalactiae* obtained the highest uptake. All yeasts tested, *C. albicans* ATCC 10231 and clinical strain (CS 2502) accumulated much more [^18^F]FDG than bacteria.

Enterobacterales (i.e., *E. coli* and *Enterobacter*) was the only group of bacteria that accumulated [^18^F]FDS (Figure 3B). The uptake values of [^18^F]FDS were at least three times lower than those of [^18^F]FDG, for example, in the reference bacteria *E. coli* ATCC 25922 ([^18^F]FDG = 605.28 ± 91.91 Bq/10^6^ CFU and [^18^F]FDS = 160.23 ± 71.76 Bq/10^6^ CFU).

All bacteria and yeasts accumulated [^18^F]FPABA (Figure 3C). There were no clear differences between GPs, GNs, yeasts, and the microaerophilic GP, *C. acnes*. The two highest accumulations were obtained in *S. aureus* CS 16557 and *E. coli* ATCC 25922. Comparison of different strains of the same species of *S. aureus* showed large differences (*S. aureus* CS 3082 = 954.29 ± 568.26 Bq/10^6^ CFU and *S. aureus* CS MR-methicillin resistant 16557 = 5,192 ± 1334.49 Bq/10^6^ CFU). Regardless of their type, the heat-killed bacteria showed virtually no uptake of the radiotracers (Figure 3).

**Figure 4:**
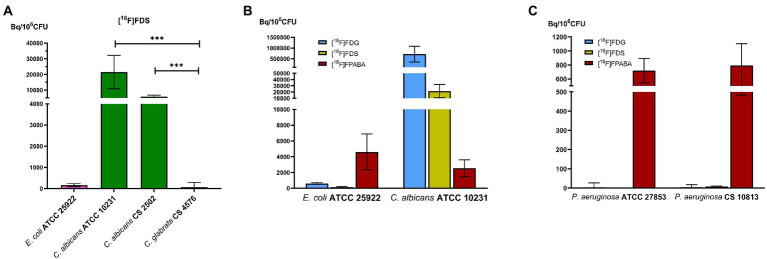
Comparison of prokaryotic and eukaryotic cells. *In vitro* experiments with *Pseudomonas aeruginosa.* **(A)** Comparison of [^18^F]FDS uptake in *Candida* species. ***p ≤ 0.001; **(B)** Cellular uptake of reference strains of bacteria and yeast by [^18^F]FDG, [^18^F]FDS, and [^18^F]FPABA; **(C)** *P. aeruginosa* uptake between [^18^F]FDG, [^18^F]FDS, and [^18^F]FPABA.

Only some yeast species accumulated [^18^F]FDS. Clinical and reference isolates of *C. albicans* took up the radiotracer, whereas *C. glabrata* did not (Figure 4A). *Candida albicans* showed more accumulation of radiolabeled monosaccharides ([^18^F]FDG and [^18^F]FDS) than bacteria. In contrast, [^18^F]FPABA accumulation was higher in *E. coli* than in fungi (Figure 4B). Interestingly, [^18^F]FPABA was the only radiotracer that was taken up by *P. aeruginosa* (Figure 4C).

The variation in the initial and final number of viable cells (CFU/ml) is shown in Supplementary Table S3. We found no relevant variation for most of the microorganisms. However, [^18^F]FDG resulted in a significant decrease in the growth of *C. acnes* (*p* ≤ 0.0001). In addition, *C. acnes* ATCC 11827, *P. aeruginosa* and *S. agalactiae* showed a non-statistically significant decrease in growth with [^18^F]FDG. Incubation with [^18^F]FDS induced a statistically significant reduction of CFU/mL in *S. agalactiae*, *C. acnes*, and *P. aeruginosa*. Only *E. coli* growth appeared to increase with incubation with [^18^F]FDS (*p* = 0.0229). [^18^F]FPABA did not change the number of bacteria or yeast in these experiments, with the sole exception of the clinical strain of *E. coli*.

### 3.3. *Escherichia coli* myositis

**Figure 5:**
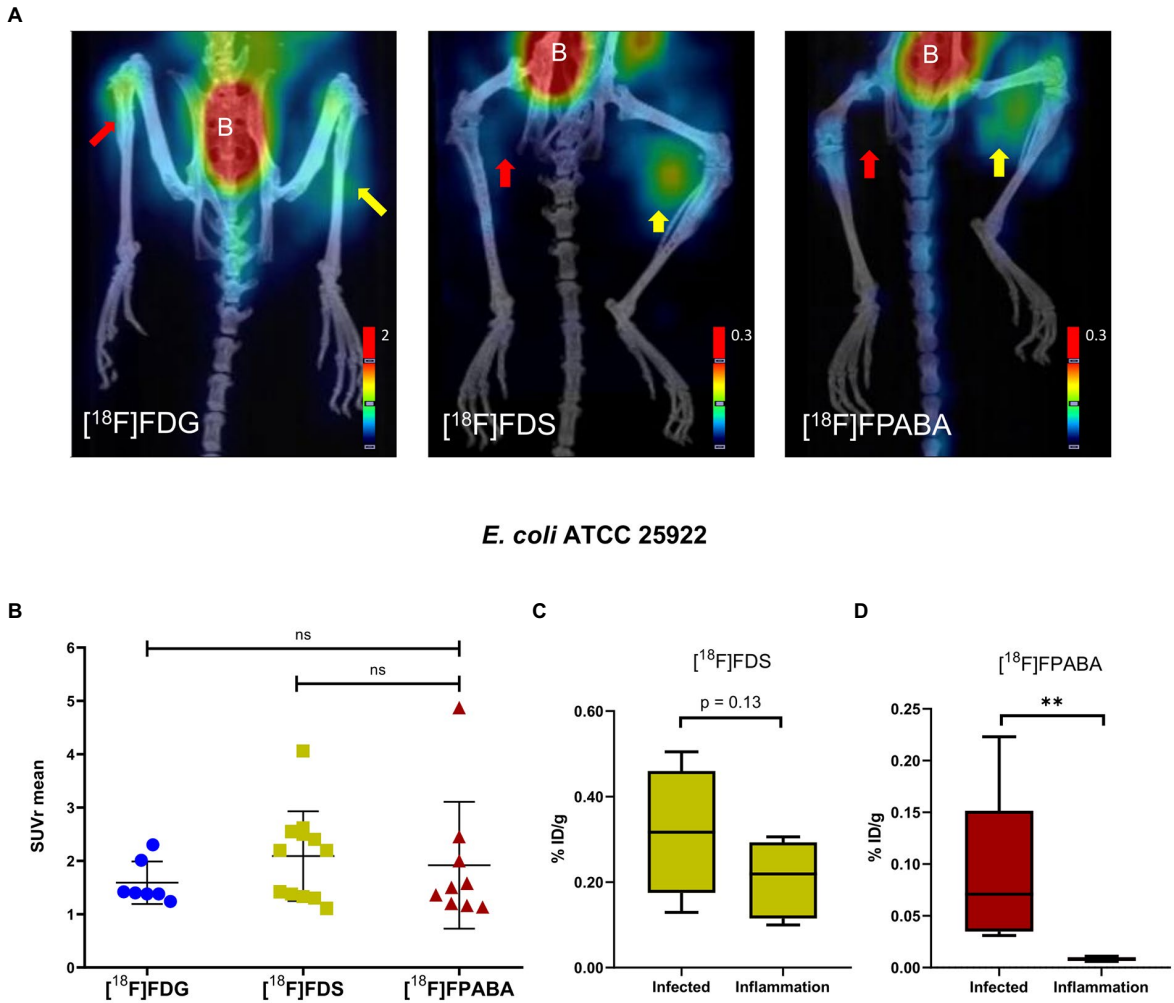
*Escherichia coli* myositis infection and inflammation of the hindlimbs. **(A)** PET/CT image of [^18^F]FDS (*n* = 12) after 120 min, [^18^F]FDG (*n* = 7), and [^18^F]FPABA (*n* = 9) after 60 min of uptake. The right limb was infected with exponentially growing bacteria (yellow arrow); the left limb was injected with heat-killed bacteria (red arrow). B = bladder. **(B)** Comparison of mean SUVr for uptake of [^18^F]FDG, [^18^F]FDS, and [^18^F]FPABA in infected and inflamed muscle tissue. Represented as mean ± SD. ns, not significant (*p* > 0.05). Kruskal-Wallis multiple-comparison test. **(C)** *Ex vivo* comparison of % injected dose of [^18^F]FDS per gram of infected versus inflamed tissue (%ID/g). **(D)** *Ex vivo* comparison of % injected dose of [^18^F]FPABA per gram of infected vs. inflamed tissue (%ID/g). **p ≤ 0.01.

The [^18^F]FDG, [^18^F]FDS, and [^18^F]FPABA PET images detected *E. coli* myositis infection (Figure 5A; Supplementary Figure S4). The SUVr values were similar for [^18^F]FDG (1.55 ± 0.39), [^18^F]FDS (2.09 ± 0.84), and [^18^F]FPABA (1.92 ± 1.19) with no statistically significant differences (Figure 5B).

The *post-mortem*  measurements of radioactivity in infected and inflamed tissues, presented as %ID/g, was not significant for [^18^F]FDS (Figure 5C). By contrast, [^18^F]FPABA readily discriminated between infection (%ID/g = 0.089 ± 0.078) and inflammation (%ID/g = 0.008 ± 0.002) and showed a statistically significant difference (*p* = 0.0079; Figure 5D).

Mean bacterial load counts at the infection site were similar, approximately 7 log_10_ across all mice and probe groups (Supplementary Table S5). In addition, strong signals with minimal background were detected at the infection site with bacterial loads of 5.59 log_10_ CFU/g with [^18^F]FDG, 5.5 log_10_ CFU/g with [^18^F]FDS, and 6.17 log_10_ CFU/g with [^18^F]FPABA.

### 3.4. *Staphylococcus aureus* myositis

**Figure 6:**
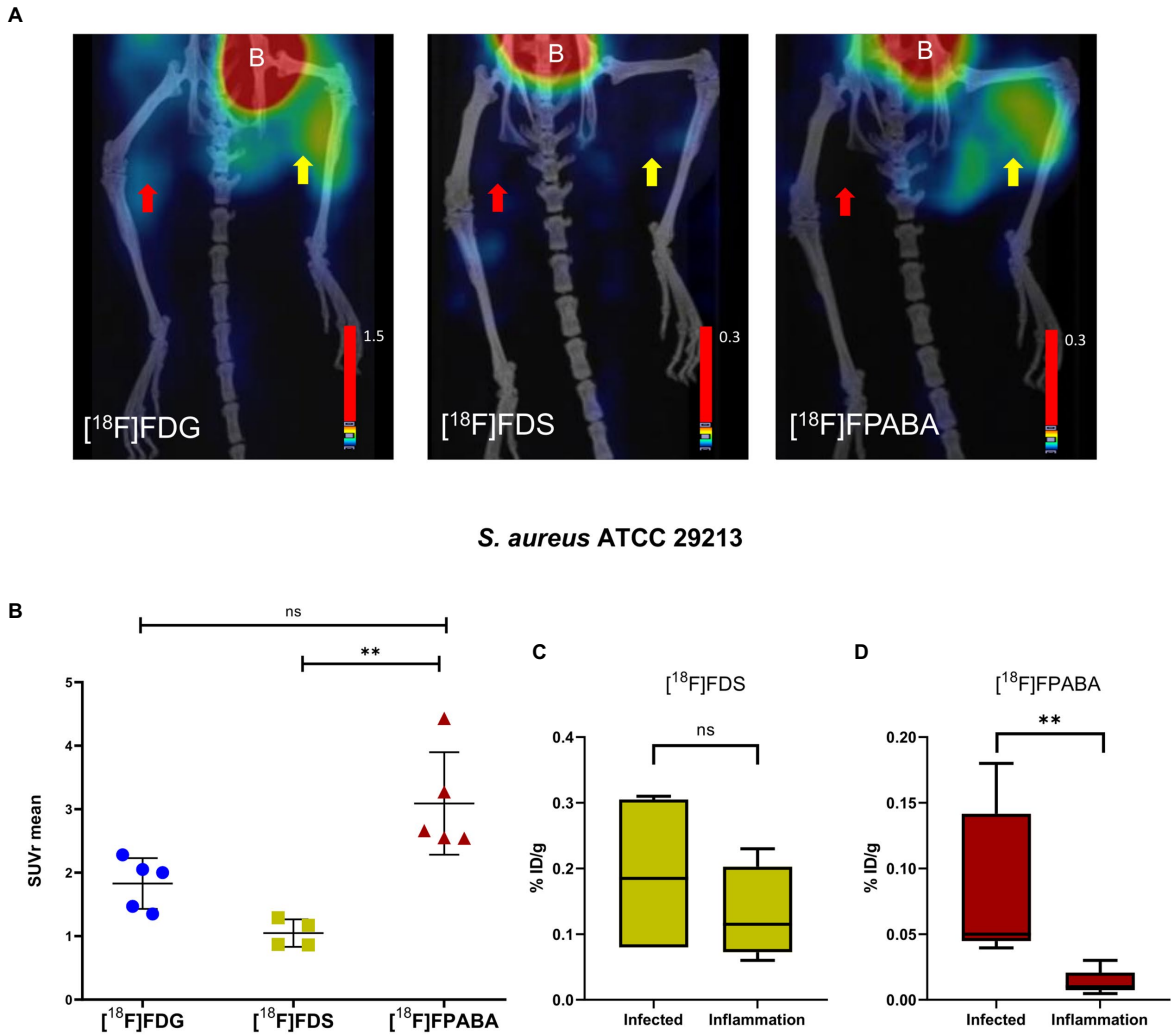
*Staphylococcus aureus* myositis infection and inflammation of hindlimbs. **(A)** PET/CT imaging with [^18^F]FDS (*n* = 4) after 120 min, [^18^F]FDG (*n* = 5), and [^18^F]FPABA (*n* = 4) after 60 min of acquisition. The right limb was infected with exponentially growing bacteria (yellow arrow); the left limb was injected with heat-killed bacteria (red arrow). B = bladder. **(B)** Comparison of mean SUVr for uptake of [^18^F]FDG, [^18^F]FDS, and [^18^F]FPABA in infected and inflamed muscle tissue. Represented as mean ± SD. ns, not significant (*p* > 0.05), *p ≤ 0.05, **p ≤ 0.01. Kruskal-Wallis multiple-comparison test. **(C)** *Ex vivo* data of % injected dose of [^18^F]FDS per gram of infected and inflamed tissue (%ID/g). **(D)** *Ex vivo* data of % injected dose of [^18^F]FPABA per gram of infected and inflamed tissue (%ID/g). **p ≤ 0.01.

[^18^F]FPABA and [^18^F]FDG detected *S. aureus* infection in the right limb, as shown in Figure 6A; Supplementary Figure S4. A non-specific [^18^F]FDG signal was observed in the inflamed muscle. As expected, the [^18^F]FDS PET images showed no uptake in the inflamed and in the infected muscle. Radiotracer uptake, expressed as SUVr, indicated that [^18^F]FPABA could distinguish infection from inflammation (SUVr = 3.090 ± 0.807), but with no statistically significant differences when compared with [^18^F]FDG (*p* = 0.1763; Figure 6B).

Analysis of *post-mortem* biopsies demonstrated a statistically significant difference between infection and inflammation with [^18^F]FPABA (*p* = 0.0079; Figure 6D).

The means of bacterial load in all experiments are shown in Supplementary Table S5. [^18^F]FDG PET and [^18^F]FPABA detected and localized *S. aureus* infection with a minimum bacterial load of 6.31 log_10_ and 7.15 log_10_ CFU/g, respectively.

## 4. Discussion

### 4.1. Contribution of different PET signals by type of microorganism: [^18^F]FDG

Our *in vitro* results expanded the number of bacteria studied (Figure 3A) compared to previous studies (Heuker et al., 2017). [^18^F]FDG uptake was higher in GPs than GNs in our model. *Streptococcus agalactiae* achieved the highest bacterial uptake, as was the case with *Streptococcus pyogenes* in other studies (Heuker et al., 2017). Since we observed almost no uptake in non-lactose fermenting gram-negative bacilli (*P. aeruginosa* and *S. maltophilia*), infections with these bacteria could be difficult to diagnose with [^18^F]FDG PET (Figure 4C). Some infectious diseases guidelines propose [^18^F]FDG PET imaging as a criterion for diagnosis (Habib et al., 2015; Haidar and Singh, 2022; Lauri et al., 2022) which may be underestimated if *P. aeruginosa* or *S. maltophilia* is the etiology of infection. These findings should be taken into account when interpreting PET images in biofilm-related infections (endocarditis associated with prosthetic valves or pacemakers). Mature biofilms are formed by stationary (dormant) bacteria that produce a minor inflammatory response. *Pseudomonas aeruginosa* is a well-known biofilm former, especially on medical surfaces, cystic fibrosis patients, or chronic wound infection. The involvement of this bacteria could reduce the negative predictive value of nuclear molecular techniques using [^18^F]FDG. There is also insufficient evidence on the sensitivity of [^18^F]FDG in non-lactose fermenters, since only case reports have reported on the use of [^18^F]FDG-PET as a diagnostic tool in *P. aeruginosa* infections (Kawamura et al., 2021). Although GP bacteria are the main biofilm producers, our data show that [^18^F]FDG uptake is higher in these microorganisms. This could increase the sensitivity for [^18^F]FDG imaging in infection related to biofilm-producing GP bacteria.

[^18^F]FDG accumulation in *Cutibacterium acnes,* which is microaerophilic and typically slow growing, was just 2 h (Figure 3A), 3 h less than the mean growth time known for this bacterium (5.1 h; Hall et al., 1994) The final CFU/ml count in the *in vitro* experiments showed a statistically significant decrease in the clinical strain of *C. acnes* and a non-significant decrease in *C. acnes* ATCC 11827. This could be explained by the fact that uptake of [^18^F]FDG by these bacteria does not require exponential growth or a suitable environment.

All yeasts tested showed higher uptake of [^18^F]FDG as compared to bacteria (Figure 3A). [^18^F]FDG could therefore be a good radiotracer for yeast infections, with the possibility of extending it to filamentous fungi. One study on the *in vitro* uptake of [^18^F]FDG showed detection of *A. fumigatus*, but unlike our experiments with yeasts, this uptake appeared to be lower than in bacteria (i.e., *E. coli*; Lai et al., 2022).

[^18^F]FDG uptake was increased in myositis infection, with nonspecific uptake in [^18^F]FDG-PET imaging of inflamed muscle tissue, notably in heat-killed *E. coli* myositis (Figures 4A, 5A). Toxin production, probably due to *S. aureus* toxins or *E. coli* endotoxins, induces elevated inflammation that generates high levels of glucose transporter expression in infection when compared with inflammation with heat-killed bacteria; this effect corrects SUV ratio values.

### 4.2. [^18^F]FDS shows acute *Escherichia coli* myositis infection and can distinguish *Candida* spp.

[^18^F]FDS was taken up *in vitro* by Enterobacterales species (i.e., *E. coli* and *E. ludwigii*), but not by GPs and non-lactose fermenters (Figure 3B), as described in other studies (Weinstein et al., 2014; Ordonez et al., 2017). *Escherichia coli* has a generation time of 84 min using ᴅ-sorbitol (Lengeler, 1975), which allowed these isolates to grow in our experiments by metabolizing [^18^F]FDS as sorbitol-6-phosphate would, generating fructose-6-phosphate in the process. Only *E. coli* ATCC 25922 showed a statistically significant increase in numbers using [^18^F]FDS (Supplementary Table S3). Future studies will be necessary therefore to confirm whether this bacterium shows growth with fluorinated sorbitol.

We also demonstrated uptake in yeasts such as *Candida albicans* (Figure 4A). The higher [^18^F]FDG uptake (3-fold) compared to [^18^F]FDS may be related to the fact that sorbitol is not the preferred carbon source in Enterobacterales and *C. albicans* (Figure 4B). In recent experiments, *E. coli* and *A. fumigatus* had a higher uptake (approximately 10-fold) with [^18^F]FDG than with [^18^F]FDS (Lai et al., 2022). A clear difference in uptake of [^18^F]FDS by *Candida glabrata* was shown (66.54 ± 224.67 Bq/10^6^ CFU) when compared with the other two strains of *C. albicans* analyzed (ATCC 10231 = 21496.06 ± 10684.79; clinical strain = 5846.24 ± 924.90 Bq/10^6^ CFU; Figure 4A). [^18^F]FDS is therefore useful to discriminate between yeasts with positive sorbitol assimilation (i.e., *C. albicans*) and those unable to assimilate sorbitol (i.e., *C. glabrata*; Kurtzman and Fell, 1998). This result could improve the diagnosis of disseminated/invasive *Candida albicans* infections (especially in the liver, CNS, eye, and other sterile locations) using [^18^F]FDS-PET. We obtained higher uptakes (Bq/10^6^ CFU) in *C. albicans* than *E. coli* ATCC 29213, as was the case in a recently published study (Kim et al., 2022). In another study however, the results showed that [^3^H]sorbitol uptake was 10-fold lower in *C. albicans* than in *E. coli* at 120 min (Lai et al., 2022). This difference in uptake probably depends on the units used for normalization. A universal indicator to normalize both bacterial and fungal biomass is challenging because of differences in growth and cell size (fungi are much larger than bacterial cells). *In vitro* uptake may be underestimated when normalized to the amount of protein (Kim et al., 2022). This discrepancy could probably be resolved in further experiments, by testing *in vivo* uptake in an animal model of acute mixed infections with *C. albicans* and *E. coli* (normalized to final CFU/g per tissue).

The [^18^F]FDS PET images clearly differentiated between inflammation and *E. coli* myositis infection although *postmortem* biodistribution studies found no uptake (%ID/g) differences between hindlimbs (Figures 5A,C). This diagnostic imaging with class-specific bacterial and yeast probes could be of interest when clinicians have a definite etiology of the infection. Even so, it is difficult to differentiate between all types of bacteria and microorganisms with probes because of the large number of different species. [^18^F]FDS is a GN-specific probe with a low limit of detection (5.5 log_10_ CFU/g) and so can diagnose chronic infections with Enterobacterales. Its previous translation to humans (Yao et al., 2016; Zhu et al., 2016; Ordonez et al., 2021) supports safe use of this technology in chronic infections. The limitation of our results is that [^18^F]FDS may not differentiate between bacteria and yeasts and so adequate clinical information is needed. It would be interesting to develop animal models (with yeasts or mixed infections with bacteria and yeasts) to check and compare [^18^F]FDS-PET signals. To avoid negative results in specific detection of Enterobacterales and *C. albicans*, sequential use of other radiotracers could be considered to broaden the range of pathogens identified. In addition, [^18^F]FDS imaging could improve the monitoring of prolonged treatment in deep-seated Enterobacterales infections, as antimicrobial therapy may fail due to the impaired drug penetration or acquisition of antimicrobial resistance.

### 4.3. New synthesis of [^18^F]FPABA: An approach to an imaging probe for all bacterial and yeast species

The proposed synthesis pathway to obtain [^18^F]FPABA offers a promising solution due to its various advantages, which include the ease of obtaining the radiosynthetic precursor, the simple intermediate steps using everyday laboratory reagents, all highly soluble in water. The simplicity of the pathway also facilitates synthesis on different automated radiosynthesizers if a semipreparative radio-HPLC system is available. Further improvements in synthesis, yet unexplored, may be able to increase the yield while maintaining the radiochemical purity of the final product above 97%. [^18^F]FPABA appears to be a better radiotracer than [^11^C]PABA, not only because of the longer half-life of the radionuclide (110 min for fluorine-18 vs. just 20 min for carbon-11), but probably also because of the *in vivo* metabolism of the radiotracer, which could complicate image interpretation in the carbon-11 tracer. The accumulation of sulfonamide-like chemicals in the cell depends to a significant extent on the degree of ionization in the cytoplasm and the surrounding medium (Zarfl et al., 2008). Zarfl et al. point out that no accumulation occurs in the cell if the external pH exceeds the intracellular pH. A lower pH amplifies microorganisms that can accumulate because most bacteria have an intracellular pH of around 7 (*E. coli* = 7.6).

*In vitro* uptake of [^18^F]FPABA was tested in all bacterial and yeast strains (Figure 3C). [^18^F]FPABA appears to be a better probe for all bacteria and yeasts for diagnosis of infection, with higher *in vitro* uptake than [^18^F]FDS. Previous *in vitro* studies have examined *S. aureus* and *E. coli* with [^18^F]FPABA and [^11^C]PABA (Zhang et al., 2018; Ordonez et al., 2022). *Pseudomonas aeruginosa* and *Mycobacterium tuberculosi*s were assessed only with ^3^H (Ordonez et al., 2017), but no other species were tested. We obtained the same promising results in Enterobacterales, *S. aureus*, and *P. aeruginosa*. New data were obtained for *C. acnes*, yeast, *S. epidermidis*, and Enterobacterales. Unlike [^18^F]FDG and [^18^F]FDS, *P. aeruginosa* incorporates [^18^F]FPABA (Figure 4C), which makes this probe of interest in nosocomial infections. Although the innovative ^68^Ga- and [^18^F]FIAU-radiolabeled siderophores showed potential in models for bacteria-specific imaging with *P. aeruginosa* (Petrik et al., 2021), they fell short when translated to human clinical trials (Zhu et al., 2016; Cho et al., 2020).

Different *in vitro* uptakes were obtained in strains of the same species (*S. aureus* and *S. epidermidis*), as was found in other studies performed with [^18^F]FPABA and *S. aureus* (Zhang et al., 2018). DHPS has different expression levels in the same bacterial species, which may explain the different results observed (Wang et al., 2021). Alterations in this enzyme, one of the mechanisms of bacterial resistance, correlate with the low effectiveness of sulfonamides in some species or strains. These resistance mechanisms or concomitant treatment prior to image acquisition may affect [^18^F]FPABA activity. In addition, AbgT transporters are exporters of PABA and interspecies variation may have an impact (Delmar and Yu, 2016). Furthermore, bacteria are capable of *de novo* PABA synthesis from chorismate and glutamine (Dosselaere and Vanderleyden, 2001). Therefore, future studies should evaluate possible signal variation in PABA PET imaging.

To the best of our knowledge, this is the first study of *in vitro* uptake of [^18^F]FDG, [^18^F]FDS, and [^18^F]FPABA in yeasts. Our results showed [^18^F]FPABA uptake in the yeasts tested and suggest that it is a promising future tool in fungal diagnostics. We suspect that these results of *in vitro* uptake are because DHFR (dihydrofolate reductase), an enzyme essential for folate-dependent pathways in fungi, is a valid antifungal target in *C. albicans* (DeJarnette et al., 2020). The DHFR enzyme is the step subsequent to PABA incorporation into DHPS. In addition, DHPS inhibitors such as sulfamethoxazole are an effective treatment for *Pneumocystis* pneumonia (caused by the fungus *Pneumocystis jirovecii*). Although folate biosynthesis pathways and enzymes have been characterized in both bacteria and plants, they are not well studied or characterized in yeasts. Our results confirm that yeast requires *in vitro* uptake of PABA and that [^18^F]FPABA could be used as a probe for yeasts in PET imaging.

[^18^F]FPABA PET, performed on mouse models of *E. coli* and *S. aureus* myositis, clearly differentiated between inflammation and infection and showed no uptake in the inflamed limb (Figures 5A, 6A). The SUVr values of [^18^F]FPABA were lower than expected (Figures 5B, 6B) because the bone showed uptake and was difficult to avoid when VOIs were drawn on the image. In *post-mortem* biodistribution studies, Zhang et al. observed that [^18^F]FPABA accumulated in the tibia at different rates (at 30, 60, and 120 min) with high ID%/g values, which might explain why some of the bone was seen on images and the SUV was less sensitive (Zhang et al., 2018). Our interpretation however was limited to visual analysis and current advances in artificial intelligence can certainly improve our image data (Schwenck et al., 2022). However, the post-mortem analysis between hindlimbs with infection (*S. aureus* and *E. coli*) and inflammation was statistically significant, and clearly demonstrated detection of infection (Figures 5C, 6C).

The mean bacterial load in acute *S. aureus* and *E. coli* myositis in our experiments (Supplementary Table S5) was around 7 log_10_ CFU/g, and [^18^F]FPABA localized all these infections. This bacterial load is low compared to acute infections, where the CFU/mL is 8 log_10_ or more (Davey and Barza, 1987; König et al., 1998). However, the infection load for chronic or bloodstream infections is even lower (Kang et al., 2014). [^18^F]FPABA uptake remains high regardless of the growth phase (Zhang et al., 2018) and testing this probe on lower bacterial loads in chronic infections and implant-associated biofilms could be of interest in the future. PET imaging with [^11^C]PABA on a rabbit model of *S. aureus* prosthetic implant infection obtained a good discrimination ratio (target-to-nontarget tissue) of 3.17 (Ordonez et al., 2022). The main problem in that study was that the image was obtained 7 days after infection and with bacterial loads of 7-log_10_ CFU, similar to acute infection (without the decreased sensitivity associated with low bacterial load). [^18^F]FPABA uptake into other typical implant-associated microorganisms, such as *C. acnes,* was *in vitro* in our study and it would be interesting to test them on *in vivo* models. It could be an improvement over microbiological tests that are long (14 days) and require complicated incubation atmospheres (microaerophilic/anaerobic; Schäfer et al., 2008).

## 5. Conclusion

[^18^F]FDG is actively incorporated by most bacteria and yeasts. Nevertheless, non-lactose fermenters (i.e., *P. aeruginosa* and *S. maltophilia*) show no uptake *in vitro* and further in-depth studies are needed to clarify this aspect, as the metabolic basis is unknown. In the future, animal models and clinical studies should focus on non-lactose fermenters to check the diagnosis of infection using [^18^F]FDG PET, even if the signal depends on other factors (such as inflammation, sensitivity, PET camera resolution, surrounding tissue, or target-to-normal tissue ratio).

[^18^F]FDS is metabolized by Enterobacterales and is able to differentiate yeasts that assimilate sorbitol (i.e., *Candida albicans*) from those that do not (i.e., *Candida glabrata*). [^18^F]FDS PET imaging could therefore be useful when there are appropriate clinical data focused on Enterobacterales infection (i.e., hepatobiliary infections, intestinal focus) or IFIs caused by *C. albicans*. Murine models of acute *E. coli* infection show nonspecific uptake of [^18^F]FDG due to sterile inflammation, but no inflammation with [^18^F]FDS.

[^18^F]FPABA appears to be a potential probe for imaging infections as all tested bacteria and yeasts showed [^18^F]FPABA uptake, even fastidious microorganisms (such as *C. acnes*). [^18^F]FPABA was the only probe tested that showed *in vitro* uptake of *P. aeruginosa,* unlike [^18^F]FDG and [^18^F]FDS. Therefore, it is crucial to know why uptake of [^18^F]FPABA is different in some bacterial species and to expand studies to correlate this with the biological characteristics of the different microorganisms. Further investigation in both *in vitro* and *in vivo* experiments is needed to select the best PET imaging probes for *P. aeruginosa*.

In acute myositis, both GP and GN bacteria incorporate [^18^F]FPABA.

The limitations of our study that should be addressed in the future include testing microorganisms that affect immunocompromised patients (i.e., *Aspergillus, P. jirovecii*), *in vivo* models with yeast species, *Corynebacterium* spp. and chronic infections. Future studies on chronic infections with low numbers of bacteria and yeast using [^18^F]FPABA could also be very interesting to further broaden the potential clinical applications of this radiotracer.

## Data availability statement

The original contributions presented in the study are included in the article/Supplementary material; further inquiries can be directed to the corresponding author.

## Ethics statement

The animal study was reviewed and approved by Ethics Committee for Animal Experimentation of the University of Navarra (protocol no. 103-17).

## Author contributions

MR, JS, MC, and IP designed and analyzed the data. MC and IP coordinated the study. JS, RR, FP, KP, JLl, and IP synthesized [18F]FDS and designed the [18F]FPABA synthesis. MR, JS, and MC performed the *in vitro* experiments. MR, ME, and FC-T conducted the animal experiments and microbiological studies. MC analyzed the animal images. MR, MC, JLe, FC-T, JD, and IP provided critical comments on this project. MR, JS, MC, and IP wrote the initial draft, and all coauthors edited the manuscript. All authors contributed to the article and approved the submitted version.

## Funding

This work was funded by research project PI17/00873 from the Spanish Ministry of Health. Part of the work was supported by MCIN/AEI/10.13039/501100011033 (PID2020-117656RB-I00).

## Conflict of interest

The authors declare that the research was conducted in the absence of any commercial or financial relationships that could be construed as a potential conflict of interest.

## Publisher’s note

All claims expressed in this article are solely those of the authors and do not necessarily represent those of their affiliated organizations, or those of the publisher, the editors and the reviewers. Any product that may be evaluated in this article, or claim that may be made by its manufacturer, is not guaranteed or endorsed by the publisher.
