# Supplementary material for: Infection-specific PET imaging with 18F-fluorodeoxysorbitol and 2-[18F]F-ρ-aminobenzoic acid: An extended diagnostic tool for bacterial and fungal diseases
Source: Front Microbiol. 2023 Jan 25;14:1094929. doi: 10.3389/fmicb.2023.1094929 (PMC9905739; doi:10.3389/fmicb.2023.1094929)
Supplement: Supplementary file 1 [file Data_Sheet_1.docx]

Supplementary Material

# Supplementary Figures and Tables

**Supplementary Table S1.** **Bacterial and yeast strains used in the *in vitro* experiments.**

| Microorganism | ATCC or clinical strain (CS) | Sample |
| --- | --- | --- |
| *S. aureus* | ATCC 29213 | - |
|  | ATCC 25923 | - |
|  | CS 3082 MSSA | Hip prosthesis |
|  | CS 16557 MRSA | Tumor abscess, mega prosthesis of tibia |
| *S. epidermidis* | ATCC 35984 | - |
|  | ATCC 12228 | - |
|  | CS 4871 | Knee prosthesis |
| *S. agalactiae* | CS 24771 | Elbow prosthesis |
| *C. acnes* | ATCC 11827 | - |
|  | CS 11471 | Hip prosthesis |
| *E. coli* | ATCC 25922 | - |
|  | CS 11789 | Aortic biopsy, Trifecta™ aortic prosthesis |
| *E. cloacae complex*  *(E. ludwigii)* | CS 11789 | Hip prosthesis |
| *P. aeruginosa* | ATCC 27853 | - |
|  | CS 10813 | Hip prosthesis |
| *S. maltophilia* | CS 17031 | Totally implantable venous access device  (PAC) |
| *C. albicans* | ATCC 10231 | - |
|  | CS 2502 | Gentamicin-cement spacer, hip |
| *C. glabrata* | CS 4576 | Kirschner wire, left foot |

MSSA= Methicillin-Susceptible *S. aureus*, MRSA= Methicillin-Resistant *S. aureus*, PAC= Port-a-Cath^®^.


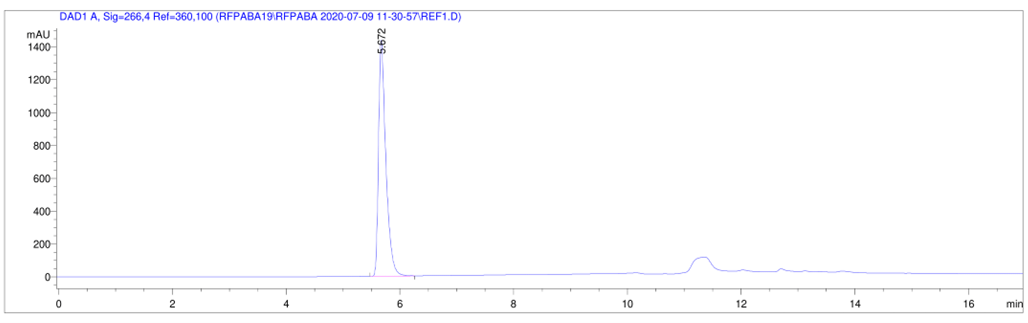

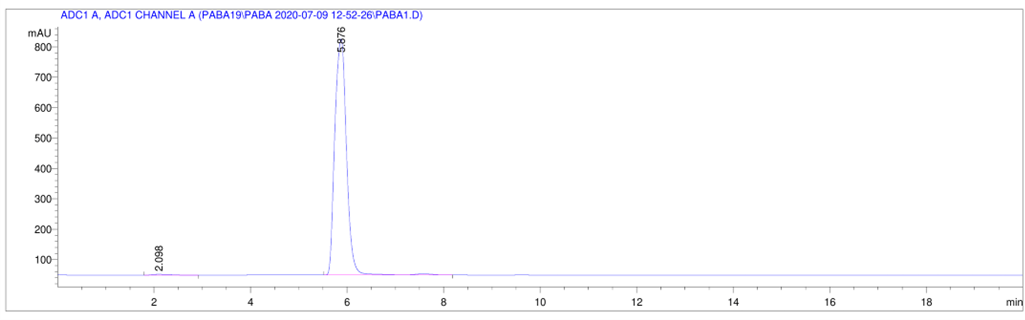

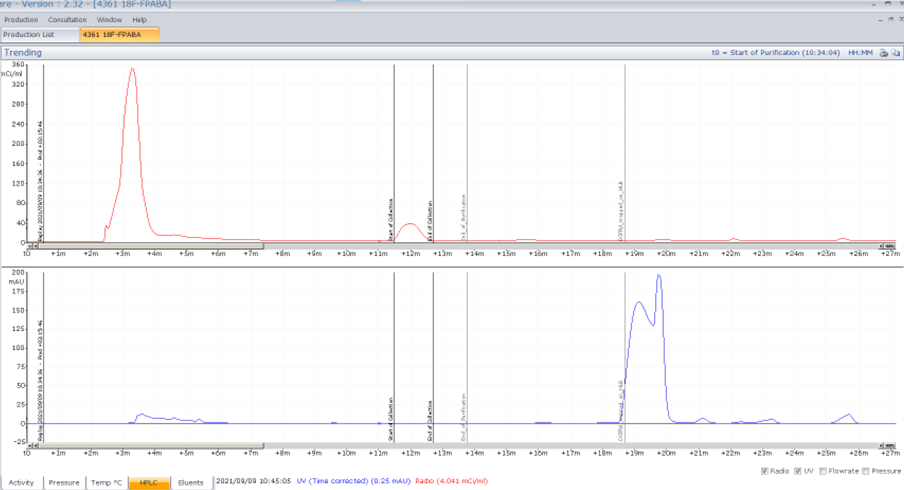


**A**

**C**

**B**

**Supplementary Figure S2. Analytical HPLC analysis of [^18^F]FPABA (A)** HPLC of a non-radioactive reference sample solution of 2-fluoro-4-aminobenzoic acid; a single peak was observed at 5.6-5.8 minutes on the UV signal chromatograph at 254 nm. **(B)** Radioactive signal from the HPLC analysis of a [^18^F]FPABA solution. The peak at 5.8 minutes was identified as the radiopharmaceutical, while the signal at 2.0 minutes corresponded to the free fluoride (< 1%). **(C)** Semipreparative radio-HPLC purification chromatogram. Data from the radioactive detector can be seen in red, while the data from the UV detector (254 nm) are shown in blue. Impurities appeared from 2.5 to 6 minutes, while the peak corresponding to [^18^F]FPABA appeared at 11.5 minutes. A high peak in the UV corresponding to the unreacted precursor can be observed.

**Supplementary Table S3.** **Bacterial** **growth on the plates at the end of *in vitro* experiments.** CFU/Bq values are compared with Sidak’s multiple comparisons test. SD= standard deviation, ND= no data, ns= not significant (p ≥0.0332), * = p<0.0332, ** = p<0.0021, *** = p<0.0002, **** = p<0.0001. Numbers in red and *p* values in green indicate a significant decrease and significant increase, respectively, in the number of colonies. The *in vitro* uptake results were converted into a qualitative variable that was compared with the results of heat-killed *E. coli* ATCC 25922 as follows: **+++** = difference of > 10^3^ Bq/10^6^, **++** =difference >100 Bq/10^6^, **+** = difference > 10 Bq/10^6^ and **-** = less than 10 Bq/10^6^.

|  |  | Initial CFU/ml (mean ± SD) | Final CFU/ml (mean ± SD) | Mean diff. | *p* value | *In vitro* uptake |
| --- | --- | --- | --- | --- | --- | --- |
| *S. aureus* ATCC 29213 | [^18^F]FDG | 8.07 ± 0.16 | 9.06 ± 0.00 | 0.99 | ns | +++ |
|  | [^18^F]FDS | 8.24 ± 0.10 | 7.56 ± 0.45 | -0.69 | ns | - |
|  | [^18^F]FPABA | 8.15 ± 0.15 | 7.85 ± 0.12 | -0.30 | ns | ++ |
| *S. aureus* ATCC 25923 | [^18^F]FDG | 7.83 ± 0.16 | 8.98 ± 0.25 | **1.15** | ******* | +++ |
|  | [^18^F]FDS | 8.21 ± 0,16 | 7.46 ± 0.72 | -0.75 | ns | - |
|  | [^18^F]FPABA | 7.83 ± 0.24 | 7.56 ± 0.10 | -0.27 | ns | +++ |
| *S. aureus* CS 3082 | [^18^F]FDG | 8.44 ± 0.19 | 9.28 ± 0.11 | **0.84** | ***** | +++ |
|  | [^18^F]FDS | 8.29 ± 0.18 | 7.52 ± 0.06 | -0.77 | ns | - |
|  | [^18^F]FPABA | 8.28 ± 0.08 | 8.96 ± 0.58 | 0.67 | ns | ++ |
| *S. aureus* CS MR 16557 | [^18^F]FDG | 8.02 ± 0.26 | 9.35 ± 0.13 | **1.33** | ******** | +++ |
|  | [^18^F]FDS | 8.20 ± 0.13 | 8.89 ± 0.10 | 0.68 | ns | - |
|  | [^18^F]FPABA | 8.34 ± 0,05 | 8.85 ± 0.30 | 0.51 | ns | +++ |
| *S. epidermidis* ATCC 35984 | [^18^F]FDG | 7.91 ± 0.13 | 8.97 ± 0.35 | **1.06** | ***** | +++ |
|  | [^18^F]FDS | 8.05 ± 0.19 | 8.23 ± 0.42 | 0.17 | ns | - |
|  | [^18^F]FPABA | 8.06 ± 0.34 | 7.99 ± 0.50 | -0.08 | ns | ++ |
| *S. epidermidis* ATCC 12228 | [^18^F]FDG | 8.02 ± 0.33 | 8.34 ± 0.67 | 0.32 | ns | +++ |
|  | [^18^F]FDS | 8.04 ± 0.00 | 8.23 ± 0.16 | 0.19 | ns | - |
|  | [^18^F]FPABA | 7.92 ± 0.02 | 7.67 ± 1.45 | -0.25 | ns | ++ |
| *S. epidermidis* CS 4781 | [^18^F]FDG | 7.91 ± 0.23 | 7.85 ± 0.09 | -0.06 | ns | +++ |
|  | [^18^F]FDS | 8.35 ± 0.08 | 8.39 ± 0.23 | 0.04 | ns | - |
|  | [^18^F]FPABA | 7.81 ± 0.32 | 8.28 ± 1.13 | 0.47 | ns | ++ |
| *S. agalactiae* CS 4771 | [^18^F]FDG | 7.33 ± 0.28 | 6.55 ± 0.58 | -0.78 | ns | +++ |
|  | [^18^F]FDS | 8.01 ± 0.61 | 6.97 ± 0.22 | **-1.13** | ****** | - |
|  | [^18^F]FPABA | ND | ND | ND | ND | ND |
| *C. acnes* ATCC 11827 | [^18^F]FDG | 6.36 ± 0.63 | 6.07 ± 0.48 | -0.29 | ns | +++ |
|  | [^18^F]FDS | 7.54 ± 0.16 | 7.33 ± 0.11 | -0.21 | ns | - |
|  | [^18^F]FPABA | 7.40 ± 0.32 | 7.64 ± 0.12 | -0.41 | ns | +++ |
| *C. acnes* CS 11471 | [^18^F]FDG | 8.33 ± 0.09 | 6.86 ± 0.42 | **-1.47** | ******** | +++ |
|  | [^18^F]FDS | 8.46 ± 0.91 | 7.24 ± 0.33 | **-1.22** | ****** | - |
|  | [^18^F]FPABA | 7.76 ± 0.29 | 7.35 ± 0.23 | 0.24 | ns | ++ |
| *E. coli* ATCC 25922 | [^18^F]FDG | 8.08 ± 0.10 | 8.87 ± 0.55 | 0.78 | ns | ++ |
|  | [^18^F]FDS | 8.19 ± 0.26 | 9.17 ± 0.21 | **0.98** | ***** | ++ |
|  | [^18^F]FPABA | 8.08 ± 0.10 | 9.00 ± 0.26 | 0.91 | ns | +++ |
| *E. coli* CS 6069 | [^18^F]FDG | 8.03 ± 0.23 | 9.28 ± 0.22 | **1.25** | ******* | +++ |
|  | [^18^F]FDS | 8.15 ± 0.17 | 8.94 ± 0.09 | 0.80 | ns | + |
|  | [^18^F]FPABA | 7.99 ± 0.16 | 9.42 ± 0.05 | **1.43** | * | +++ |
| *E. ludwigii* CS 11789 | [^18^F]FDG | 8.08 ± 0.28 | 8.84 ± 0.01 | 0.76 | ns | +++ |
|  | [^18^F]FDS | 8.26 ± 0.08 | 9.12 ± 0.54 | 0.86 | ns | + |
|  | [^18^F]FPABA | 8.32 ± 0.09 | 8.72 ± 0.88 | 0.40 | ns | ++ |
| *P. aeruginosa* ATCC 27853 | [^18^F]FDG | 8.03 ± 0.05 | 7.34 ± 0.35 | -0.69 | ns | - |
|  | [^18^F]FDS | 8.16 ± 0.25 | 6.86 ± 0.21 | **-1.30** | ******* | - |
|  | [^18^F]FPABA | 8.26 ± 0.09 | 7.06 ± 0.35 | -1.20 | ns | ++ |
| *P. aeruginosa* CS 10813 | [^18^F]FDG | 8.24 ± 0.24 | 7.72 ± 0.12 | -0.51 | ns | - |
|  | [^18^F]FDS | 8.13 ± 0.10 | 7.64 ± 0.22 | -0.49 | ns | - |
|  | [^18^F]FPABA | 8.44 ± 0.20 | 7.89 ± 0.28 | -0.56 | ns | ++ |
| *S. maltophilia* CS 17031 | [^18^F]FDG | 8.46 ± 0.42 | 8.49 ± 0.55 | 0.02 | ns | - |
|  | [^18^F]FDS | 8.54 ± 0.40 | 8.35 ± 0.39 | -0.19 | ns | - |
|  | [^18^F]FPABA | ND | ND | - | - | ND |
| *C. albicans* ATCC 10231 | [^18^F]FDG | 6.57 ± 0.11 | 7.58 ± 0.19 | **1.02** | ****** | +++ |
|  | [^18^F]FDS | 6.07 ± 0.13 | 5.14 ± 0.82 | **-0.93** | ***** | +++ |
|  | [^18^F]FPABA | 7.89 ± 0.28 | 5.83 ± 1.14 | **-2.06** | *** | +++ |
| *C. albicans* CS 2502 | [^18^F]FDG | 6.44 ± 0.22 | 7.02 ± 0.03 | 0.58 | ns | +++ |
|  | [^18^F]FDS | 6.55 ± 0.24 | 6.96 ± 0.45 | 0.41 | ns | +++ |
|  | [^18^F]FPABA | 6.27 ± 0.33 | 7.26 ± 0.66 | 0.99 | ns | +++ |
| *C. glabrata* CS 4576 | [^18^F]FDS | 6.89 ± 0.30 | 6.73 ± 0.49 | -0.16 | ns | + |


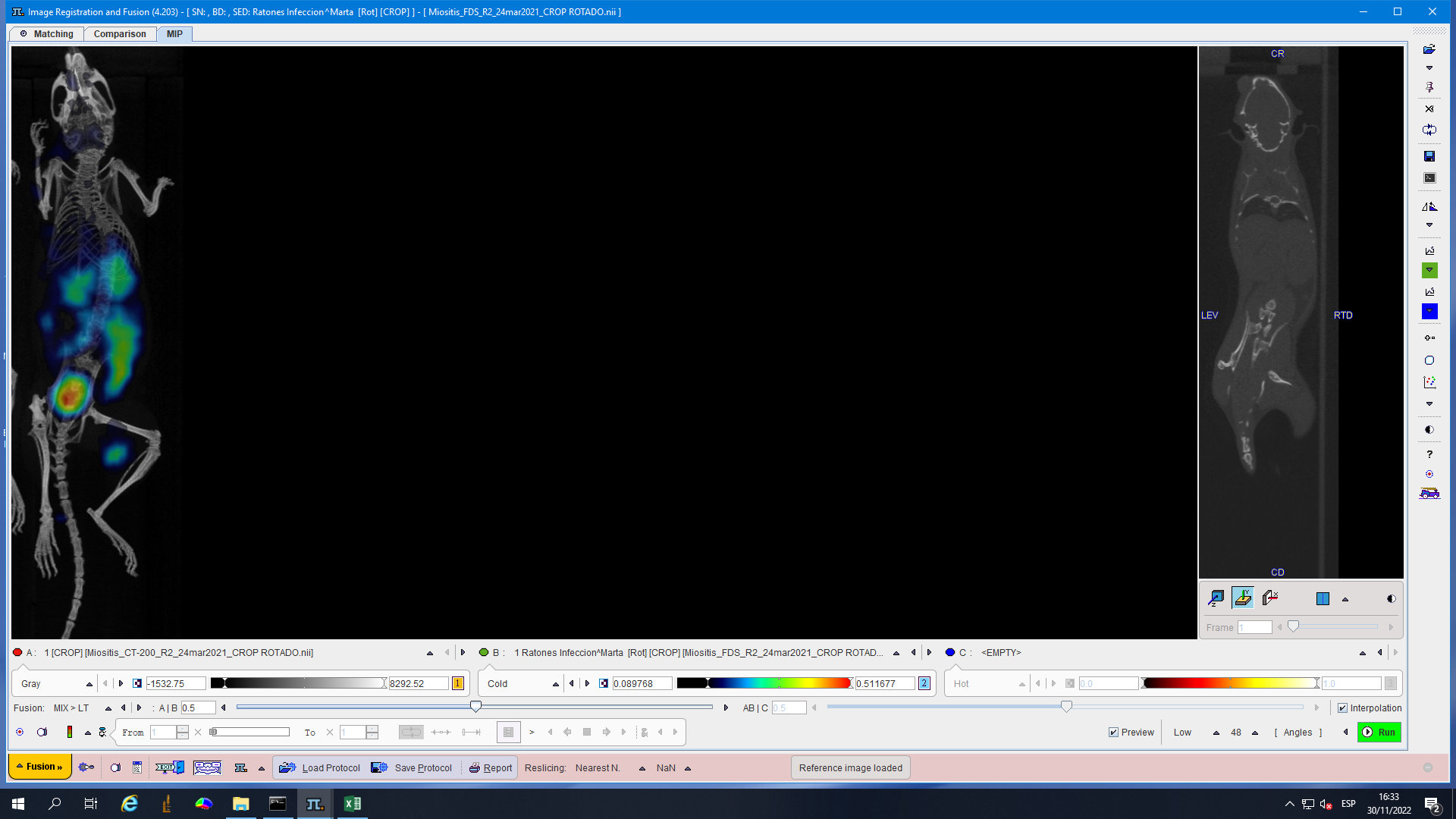

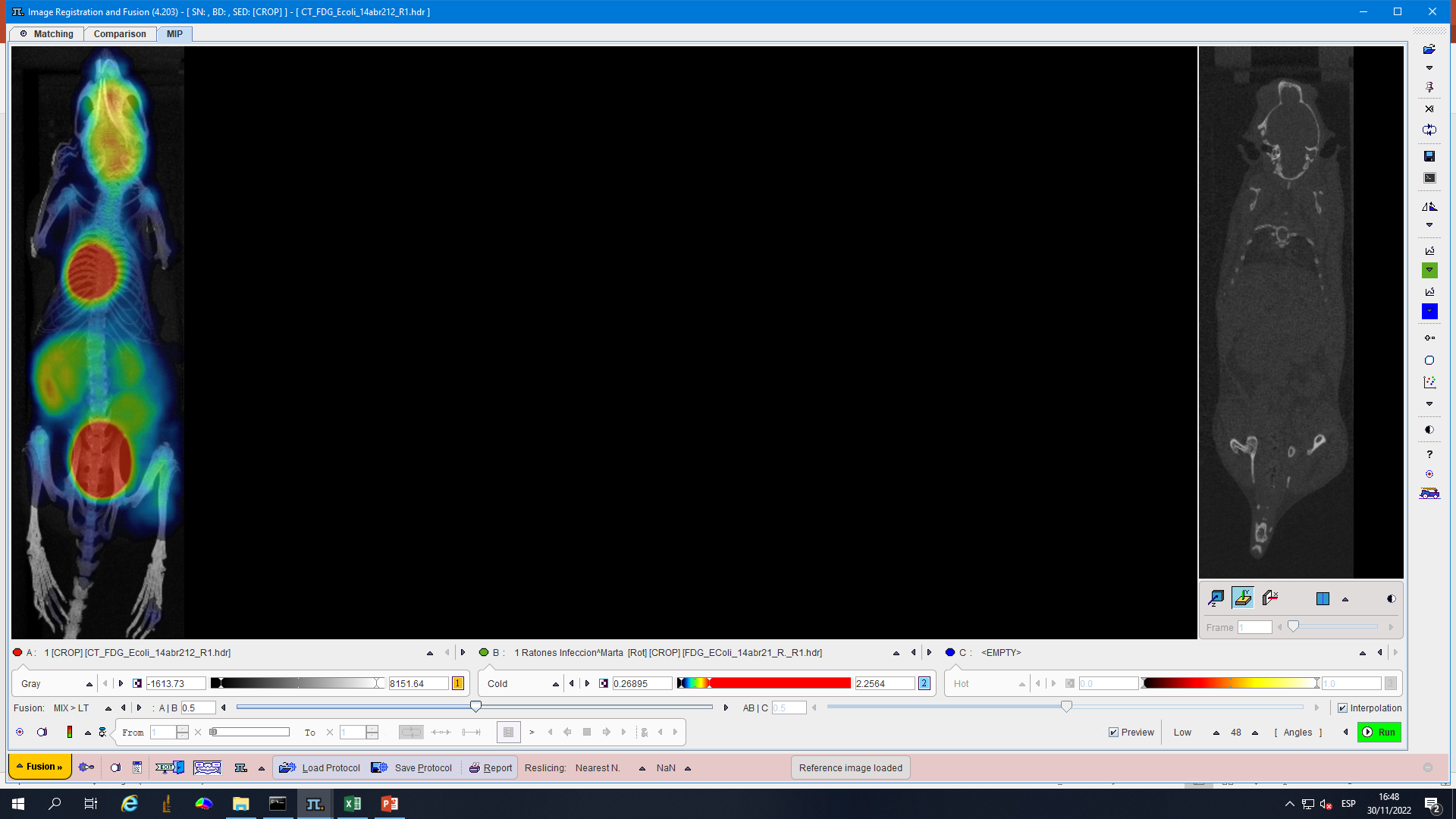

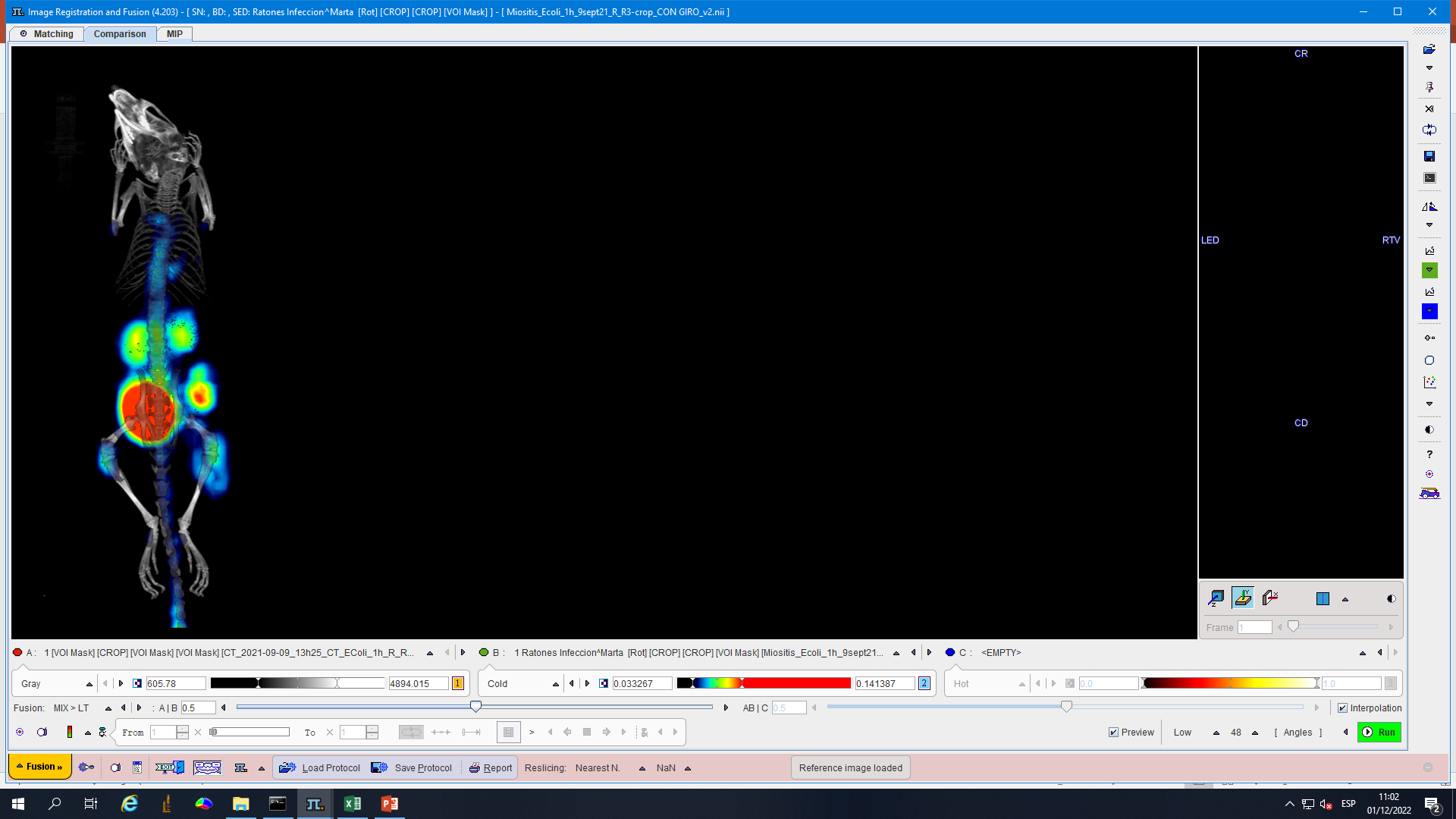


[^18^F]FPABA

[^18^F]FDG

[^18^F]FDS

li

li

k

B

B

B

h

b

k

i

i

*E. coli*

ATCC 25922

*E. coli*

ATCC 25922

*S. aureus*

ATCC 29213

k


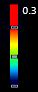

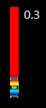


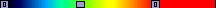


2

**Supplementary Figure S4. Biodistribution of radiotracers by PET/CT imaging.** Whole-body mouse PET/CT using [^18^F]FDG (*E. coli* ATCC 25922), [^18^F]FDS (*E. coli* ATCC 25922) and [^18^F]FPABA (*S. aureus* ATCC 29213). Infection (yellow arrow), inflammation (red arrow), brain (b), intestine (i), large intestine (li), kidneys (k), bladder (B) and heart (h). [^18^F]FDG (or its metabolites) are detected in brain (b), intestine (i), kidneys (k) and bladder (B), and also in myocardium (h: heart). In the case of [^18^F]FDS and [^18^F]FPABA, radioactivity can be observed in kidneys and bladder (showing the excretion of the radiotracers) and also in the gut, mainly in large intestine (li). This signal could be due to the specific uptake of bacteria in this location.

**Supplementary Table S5.** **Bacterial load in mice infected with myositis.** Log_10_ CFU/g at the end of the experiment (mean ± SD).

|  | *E. coli* ATCC 25922 | *S. aureus* ATCC 29213 |
| --- | --- | --- |
| [^18^F]FDG (n=12) | 7.34 ± 1.04 | 7.39 ± 0.69 |
| [^18^F]FDS (n=16) | 7.30 ± 1.04 | 7.06 ± 1.23 |
| [^18^F]FPABA (n=14) | 7.70 ± 0.94 | 7.92 ± 0.84 |
